# Prognostic Implications of Codon-Specific *KRAS* Mutations in Localized and Advanced Stages of Pancreatic Cancer

**DOI:** 10.1200/PO-25-00115

**Published:** 2026-02-19

**Authors:** Sara Raji, Hamed Zaribafzadeh, Tyler Jones, Elishama Kanu, Kunling Tong, Ashley Fletcher, T. Clark Howell, Shannon J. McCall, Jeffrey R. Marks, Bruce Rogers, Donna Niedzwiecki, Peter J. Allen, Daniel P. Nussbaum, Zahra Kabiri

**Affiliations:** ^1^ Department of Surgery, Duke University School of Medicine, Durham, NC; ^2^ Department of Biostatistics and Bioinformatics, Duke University School of Medicine, Durham, NC; ^3^ Department of Pathology, Duke University School of Medicine, Durham, NC

## Abstract

**PURPOSE:**

Although *KRAS* mutations represent the primary oncogenic driver in pancreatic ductal adenocarcinoma (PDAC), the association between codon-specific alterations and patient outcomes remains poorly elucidated, largely because of a lack of data sets coupling genomic profiling with rich clinical annotations across disease stages.

**MATERIALS AND METHODS:**

We used American Association for Cancer Research's GENIE Biopharma Consortium Pancreas v1.2 data set to test the association of codon-specific *KRAS* mutations with clinicogenomic features and patient outcomes in patients with PDAC diagnosed with localized (stages I to III) and advanced disease (stage IV). Overall survival (OS) was compared using Kaplan-Meier and multivariable Cox proportional hazards methods.

**RESULTS:**

Among 1,032 eligible patients, 949 (92%) exhibited mutant *KRAS*. These mutations were predominantly observed at G12D (n = 390, 41%), G12V (n = 305, 32%), and G12R (n = 149, 16%). In the group of patients who presented with localized disease, those with G12V mutation had notably longer survival compared with G12D mutation (*P* = .03). By contrast, patients with G12V mutation who presented with metastatic disease experienced shorter OS compared with those with G12R (*P* = .04) and G12D mutations (*P* = .04). Furthermore, no significant differences were observed in the frequencies of coaltered driver genes, including *TP53*, *CDKN2A*, and *SMAD4*, across the different *KRAS* mutations.

**CONCLUSION:**

These findings demonstrated that codon-specific *KRAS* mutations affect PDAC outcomes differently based on disease stage at diagnosis. As studies testing *KRAS* inhibitors continue to emerge and mature, the prognostic variability of individual *KRAS* mutations must be carefully considered to avoid confounding and ensure accurate evaluation of therapeutic efficacy in early-phase studies.

## INTRODUCTION

Pancreatic ductal adenocarcinoma (PDAC) has a 5-year survival rate of approximately 10%, mainly because of the lack of effective methods for early detection and treatment.^1,2^ Over 80% of patients present with advanced disease at the time of diagnosis, and outside of clinical trials, standard treatment is cytotoxic chemotherapy that provides limited benefit.^3,4^ In patients diagnosed with localized disease, the combination of surgical resection and systemic chemotherapy can improve survival in select patients; however, most patients treated with curative intent develop disease recurrence.^5^ These dismal outcomes highlight the need for improved understanding of the biologic underpinnings of PDAC.

**CONTEXT**

**Key Objective**
To evaluate the prognostic significance of codon-specific *KRAS* mutations in patients with pancreatic ductal adenocarcinoma (PDAC) diagnosed at localized and advanced stages.
**Knowledge Generated**
Using the American Association for Cancer Research GENIE Biopharma Consortium Pancreas data set, which includes genomic and clinical data from over 1,000 patients, we analyzed survival outcomes by *KRAS* mutations. In localized disease, the G12V mutation was associated with significantly longer survival compared with G12D. However, in metastatic cases, G12V was linked to worse outcomes than G12D or G12R, revealing a reversal in its prognostic role depending on disease stage.
**Relevance**
As *KRAS*-targeted therapies enter clinical trials, understanding the distinct behavior of codon-specific mutations becomes increasingly important. In particular, the paradoxical prognostic impact of G12V, found in more than one third of PDAC patients, may confound treatment efficacy assessments and should be carefully considered in trial design, data interpretation, and outcome analysis.

*KRAS* is mutated in over 90% of all patients with PDAC, serving as the primary oncogenic driver in this cancer. These *KRAS* mutations predominantly occur at codon 12, with less frequent mutations at other codons. By far, the most common codon-specific mutations are G12D (approximately 40%), G12V (approximately 30%), G12R (approximately 15%), and Q61 (H, K, L, R; approximately 6%).^6,7^ In preclinical studies, the various codon-specific *KRAS* mutations have demonstrated unique biochemical properties, suggesting that they may similarly promote differential clinicopathologic features in patients.^8,9^ Decades of research on the prognostic role of specific *KRAS* mutations in various cancers have significantly deepened our understanding of their importance in predicting clinical outcomes.^10-13^ However, results remain conflicting regarding their prognostic role in pancreatic cancer, largely because of a lack of large data sets that couple genomic profiling with rich clinical annotations.^6,7,14-19^ Additionally, previous studies have typically focused on either early or late stages of the disease, rather than across all stages.

The American Association for Cancer Research (AACR's) Project GENIE (Genomics Evidence Neoplasia Information Exchange) is a publicly accessible cancer registry that includes robust clinicogenomic data from multiple cancer centers. In this study, we conducted a retrospective analysis to elucidate the impact of codon-specific mutations on PDAC patient outcomes across disease stages, leveraging the detailed demographic-, clinical-, and treatment-level annotations which have not previously been available in other genomic profiling efforts.

## MATERIALS AND METHODS

### Patient Selection

We used the Pancreas v1.2-consortium multi-institutional and multilayer database from the AACR's GENIE Biopharma Consortium (BPC).^20^ The GENIE BPC PANC cohort comprises 1,130 samples obtained from 1,109 patients as previously reported.^21,22^ Samples with cancer types other than pancreatic adenocarcinoma, missing *KRAS* mutation data, those with the co-occurrence of *KRAS* mutations in the different categories were excluded (Data Supplement, Fig S1).

### Clinical and Genomic Data

Clinical data were curated using the previously described PRISSMM framework.^23^ Patients were categorized on the basis of stage at diagnosis: localized (stages I to III) and metastatic (stage IV). The mutation data were extracted from four centers through Synapse using the cBioPortal interface. Sequencing details can be found in the GENIE data guide (GENIE 15.1 public release). This cohort included genomic data on 39 genes that were assessed in all oncopanels. Copy number variation (CNVs) data for these genes were available for 975 patients. The term altered was used for the tumor suppressor genes *CDKN2A*, *SMAD4*, and *TP53* if any copy number loss or mutations were observed. We calculated the genomic alterations based on changes in CNVs or mutations in 35 nondriver genes, excluding *KRAS*, *CDKN2A*, *SMAD4*, and *TP53*. The Data Supplement (Table S1) lists all gene panels.

### Statistical Analyses

Descriptive statistics were used to summarize baseline patient and clinical characteristics. Comparisons between groups were performed using Chi-square or Fisher exact tests for categorical variables and the Wilcoxon rank-sum test for nonparametric continuous variables. False discovery rate correction was applied for multiple comparisons where appropriate. Survival probabilities were estimated using the Kaplan-Meier method, and differences in survival were compared using the log-rank test. Overall survival (OS) was defined as the time from initial diagnosis to either the date of death or last known follow-up, and patients alive at the last follow-up were considered censored in the analysis. The Cox proportional hazards model was used to assess prognostic factors and estimate hazard ratios (HRs) across codon-specific *KRAS *mutations while controlling for clinical features.

All statistical analyses were two-sided, with a *P* value <.05 considered statistically significant. Analyses were performed using IBM SPSS software, v28.0.1.1, GraphPad Prism, v10.2.2, and Python.

## RESULTS

### Patient Characteristics

A total of 1,032 patients were included, consisting of 464 female (45%) and 568 male (55%) patients, with a median age of 65 (IQR, 57-72) years. Patients data were derived from four centers: Memorial Sloan Kettering (MSK, 500, 48%), Dana Farber Cancer Institute (DFCI, 421, 40%), Vanderbilt Ingram Cancer Center (VICC, 63, 6.1%), and University Health Network (UHN, 57, 5.5%). Patients were relatively evenly split between those diagnosed with localized disease (565, 55%) and those diagnosed with metastatic disease (467, 45%). Complete baseline patient characteristics stratified by stage at diagnosis are listed in Table 1. *KRAS* mutations were present in 949 patients (92%) and absent in 83 patients (8%). Among the *KRAS* mutations, the most common was G12D, found in 390 cases (37.8%), followed by G12V in 305 cases (29.6%), G12R in 149 cases (14.4%), Q61 (H, R, K, and L) in 74 cases (7.2%), and other less frequent mutations in 31 cases (3%). A detailed list of *KRAS* mutations is provided in the Data Supplement (Table S2). Baseline patient characteristics according to codon-specific *KRAS* mutations are shown in Table 2.

**TABLE 1.** GENIE Cohort Patient Characteristics

Characteristic	Overall (N = 1,032)	Stages I to III (n = 565), No. (%)	Stage IV (n = 467), No. (%)
Age, years (Q1-Q3)	65 (57-72)	66 (58-72)	64 (57-71)
Sex, No. (%)			
Female	464 (45)	263 (47)	201 (43)
Male	568 (55)	302 (53)	266 (57)
Race/ethnicity, No. (%)			
Asian	37 (3.6)	16 (2.8)	21 (4.5)
Hispanic/Latinx	85 (8.2)	48 (8.5)	37 (7.9)
Non-Hispanic Black	36 (3.5)	21 (3.7)	15 (3.2)
Non-Hispanic White	837 (81)	463 (82)	374 (80)
Other	37 (3.6)	17 (3)	20 (4.3)
Center, No. (%)			
DFCI	412 (40)	212 (38)	200 (43)
MSK	500 (48)	287 (51)	213 (46)
UHN	57 (5.5)	32 (5.7)	25 (5.4)
VICC	63 (6.1)	34 (6.0)	29 (6.2)
*KRAS* status, No. (%)			
Wild type	83 (8)	44 (7.8)	39 (8.4)
G12D	390 (37.8)	214 (37.9)	176 (37.7)
G12V	305 (29.6)	172 (30.4)	133 (28.5)
G12R	149 (14.4)	85 (15)	64 (13.7)
Q61	74 (7.2)	32 (5.7)	42 (9)
Other	31 (3)	19 (3.4)	14 (3)
PDAC driver genes, No. (%)			
*TP53*			
Altered	759 (73.5)	402 (71.2)	357 (76.4)
Unknown	57 (5.5)	32 (5.7)	25 (5.4)
*CDKN2A*			
Altered	402 (39)	172 (30.4)	230 (49.2)
Unknown	57(5.5)	32 (5.7)	25 (5.4)
*SMAD4*			
Altered	379 (36.7)	183 (32.4)	196 (42)
Unknown	57(5.5)	32 (5.7)	25 (5.4)

Abbreviations: DFCI, Dana Farber Cancer Institute; MSKCC, Memorial Sloan Kettering Cancer Center; PDAC, pancreatic ductal adenocarcinoma; UHN, Princess Margaret Cancer Centre-University Health Network; VICC, Vanderbilt Ingram Cancer Center.

**TABLE 2.** Baseline Patient Characteristics According to Codon-Specific *KRAS* Mutations

Variable	G12D (n = 390)	G12V (n = 305)	G12R (n = 149)	Q61 (n = 74)	Other (n = 32)	WT (n = 83)	*P*
Age, years, No. (%)							<.007*
<65	210 (53.8)	133 (43.6)	61 (40.9)	26 (35.1)	11 (35.5)	54 (65.1)	
≥65	180 (46.2)	172 (56.4)	88 (59.1)	48 (64.9)	20 (64.5)	29 (34.9)	
Sex, No. (%)							.084
Male	204 (52.3)	183 (60)	72 (48.3)	37 (50)	18 (58.1)	54 (65.1)	
Female	186 (47.7)	122 (40)	77 (51.7)	37 (50)	13 (41.9)	29 (34.9)	
Race, No. (%)							.053
White	330 (84.6)	245 (80.3)	118 (79.2)	63 (85.1)	24 (77.4)	57 (68.7)	
Other	60 (15.4)	60 (19.7)	31 (20.8)	11 (14.9)	7 (22.6)	26 (31.3)	
Center, No. (%)							.007*
DFCI	164 (42.1)	113 (37.0)	59 (39.6)	30 (40.5)	14 (45.2)	32 (38.6)	
MSK	190 (48.7)	158 (51.8)	72 (48.3)	37 (50.0)	11 (35.5)	32 (38.6)	
UHN	12 (3.1)	16 (5.2)	13 (8.7)	2 (2.7)	1 (3.2)	13 (15.7)	
VICC	24 (6.2)	18 (5.9)	5 (3.4)	5 (6.8)	5 (16.1)	6 (7.2)	
Stage at diagnosis, No. (%)							.4
I to III	214 (54.9)	172 (56.4)	85 (57)	32 (43.2)	18 (58.1)	44 (53)	
IV	176 (45.1)	133 (43.6)	64 (43)	42 (56.8)	13 (41.9)	39 (47)	
Metastasis, No. (%)							
Liver	276 (70.8)	216 (70.8)	88 (59.1)	53 (71.6)	18 (58.1)	59 (71.1)	.07
Thorax	160 (41)	127 (41.6)	57 (38.3)	27 (36.5)	11 (35.5)	41 (49.4)	.5

Abbreviations: DFCI, Dana Farber Cancer Institute; MSKCC, Memorial Sloan Kettering Cancer Center; UHN, Princess Margaret Cancer Centre-University Health Network; VICC, Vanderbilt Ingram Cancer Center; WT, wild-type.

*P < .05.

### Genomic Landscape of PDAC in the GENIE Data Set

**FIG 1.**
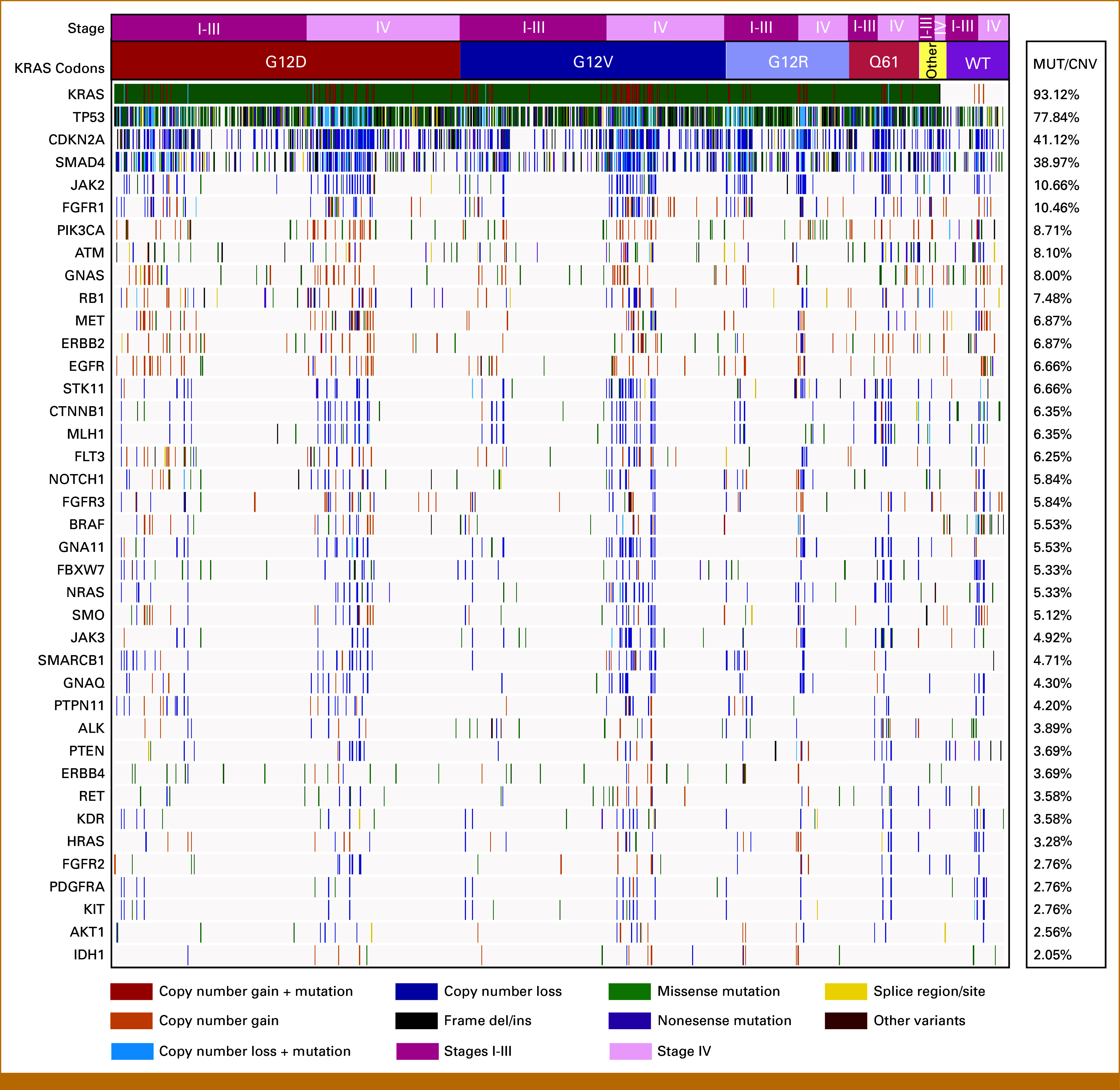
Genomic landscape of GENIE BPC PANC cohort. The oncoplot illustrates genomic alterations in 39 genes in the GENIE data set per patient for 975 patients. The distribution of codon-specific *KRAS* mutations across patients with stages I to III and stage IV PDAC are shown at the top. The right panel displays the frequency of CNVs or mutations in the cohort. The specific types of mutations as well as copy number loss and gain in the oncoplot are listed at the bottom. BPC, Biopharma Consortium; CNV, copy number variation; PDAC, pancreatic ductal adenocarcinoma.

To investigate the genomic alterations, we focused on a panel of 39 genes that had complete somatic mutation and CNV data across all four centers. Other than *KRAS*, the most commonly altered genes were *TP53* in 759 (73.5%), *CDKN2A* in 402 (39%), and *SMAD4* in 379 (36.7%). The genomic landscape, stratified by stage at diagnosis and codon-specific *KRAS* mutations, are summarized in Figure 1.

Given that PDAC tumors often exhibit co-occurring alterations in these driver genes, we next assessed the frequency of genomic alterations in *TP53*, *CDKN2A*, and *SMAD4* in the context of wild-type and mutant *KRAS* (Data Supplement, Figs S2A and S2B)*,* as well as the most common codon-specific *KRAS* mutations (Data Supplement, Fig S2C-S2F). Compared with patients with wild-type *KRAS*, patients with *KRAS* mutations were more likely to harbor co-occurring alterations in *TP53* (80.3% *v* 45.7%; *P* < .001), *TP53* and *CDKN2A* (36.6% *v* 14.2%; *P* < .001), *TP53* and *SMAD4* (33% *v* 12.8%; *P* = .003), as well as alterations in *TP53*, *SMAD4*, and *CDKN2A (*20% *v* 7.1%; *P* = .004). The absence of any of these coalterations was less common in patients with mutant *KRAS* compared with wild-type *KRAS* (10.4% *v* 27.4%; *P* = .003). Notably, no significant differences in coalterations were observed between the codon-specific *KRAS* mutations (all *P* > .05).

Moreover, alterations in *TP53*, *CDKN2A*, and *SMAD4* genes, independent of *KRAS* mutation status, had a negative prognostic impact on survival (Data Supplement, Figs S3A-S3D). In patients with *KRAS*-mutant tumors, we observed that the absence of alteration in *TP53*, *CDKN2A*, and *SMAD4* was associated with the best survival (28.3 months), whereas patients with tumors containing just one of these altered tumor suppressors had significantly reduced OS. Notably*,* the worst survival outcomes were observed in patients with tumors that harbored altered *CDKN2A*, with similar survival whether CDKN2A was altered alone (15.55 months), concurrently with *SMAD4* (14.4 months), or concurrently with *TP53* (14 months; Data Supplement, Fig S3E and Table S3). Thus, in patients with *KRAS*-mutant PDAC, loss of *CDKN2A* appears to confer a particularly dismal prognosis.

### Association of Codon-Specific *KRAS* Mutations With OS in Localized and Advanced Disease Stages

**FIG 2.**
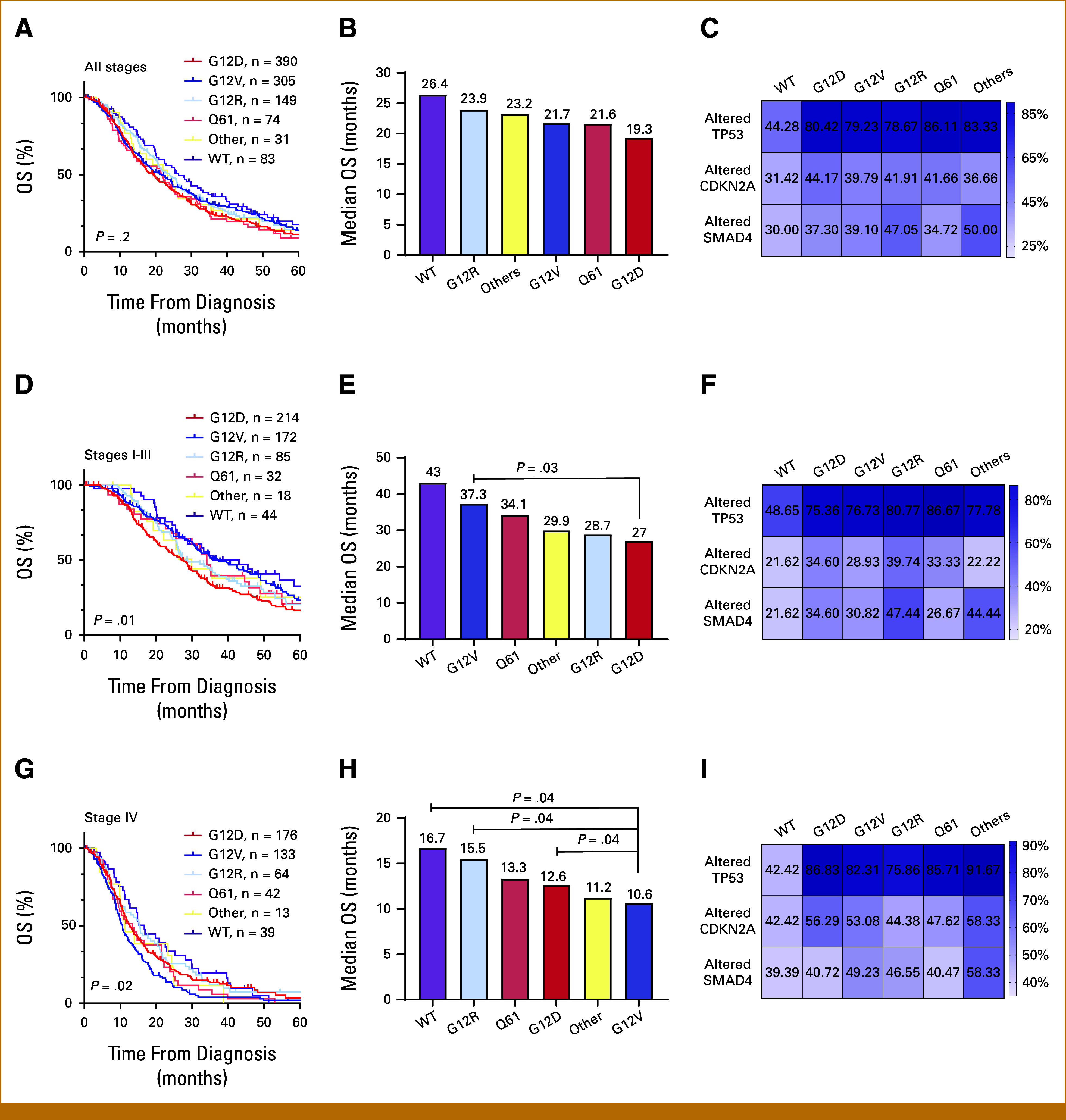
Association of *KRAS* mutations with OS. OS analyses comparing all codon-specific *KRAS* mutations in (A) all stages, (D) stages I to III, and (G) stage IV of patients with PDAC. Bar charts show the median OS of different codon-specific *KRAS* mutations and *KRAS WT* (wild-type) in (B) all stages, (E) stages I to III, and (H) stage IV of patients with PDAC. Survival was analyzed using the Kaplan-Meier method and compared between groups using the log-rank test. Heatmaps describe the frequency distribution of the codon-specific *KRAS* mutations (G12D, G12V, G12R, Q61, and other) and wild-type *KRAS* among patients with PDAC of all stages (C), stages I to III (F), and stage IV (I) with commonly coaltered tumor suppressor genes (*TP53*, *CDKN2A*, and *SMAD4*). FDR correction was applied for multiple comparisons, with a significance threshold of 0.05. The detailed information is provided in the Data Supplement (Table S4). FDR, false discovery rate; OS, overall survival; PDAC, pancreatic ductal adenocarcinoma; WT, wild-type.

Next, we investigated whether different *KRAS* mutations have distinct impacts on OS. In the main cohort (not stratified by stage), there were no statistically significant differences in OS based on codon-specific *KRAS* mutations (*P* = .2; Figs 2A and 2B), nor were the frequencies of coalterations in the common tumor suppressor genes significantly different among the codon-specific *KRAS* mutation groups (Fig 2C and Data Supplement, Table S4). Interestingly, however, codon-specific *KRAS* mutations were associated with distinct survival outcomes in patients diagnosed with localized (Figs 2D and 2E) compared with metastatic disease (Figs 2G and 2H), suggesting a prognostic role for these mutations based on disease stage.

In patients diagnosed with stage I to III disease, the G12V mutation was associated with significantly longer survival compared with the G12D mutation (37.3 months *v* 27 months; HR, 0.69 [95% CI, 0.55 to 0.87]; *P* = .03; Fig 2E). We investigated whether this difference in survival could be explained by differential frequencies of *TP53*, *CDKN2A*, and *SMAD4* coalterations among codon-specific *KRAS* mutations. However, these coalterations were not significantly different among the codon-specific *KRAS* mutation groups (Fig 2F and Data Supplement, Table S5).

Conversely, in patients diagnosed with stage IV disease, the G12V mutation was associated with shorter survival (10.6 months) compared with G12D (12.6 months; HR, 1.37 [95% CI, 1.07 to 1.75]; *P* = .04) and G12R (15.5 months; HR, 1.54 [95% CI, 1.13 to 2.10]; *P* = .04) mutations (Fig 2H). Similar to all stages and stages I to III, no significant differences were observed for the frequencies of tumor suppresser alternations among codon-specific *KRAS* mutations (Fig 2I and Data Supplement, Table S6).

### Paradoxical Impact of *KRAS* G12V on Survival on the Basis of Stage at Diagnosis

**FIG 3.**
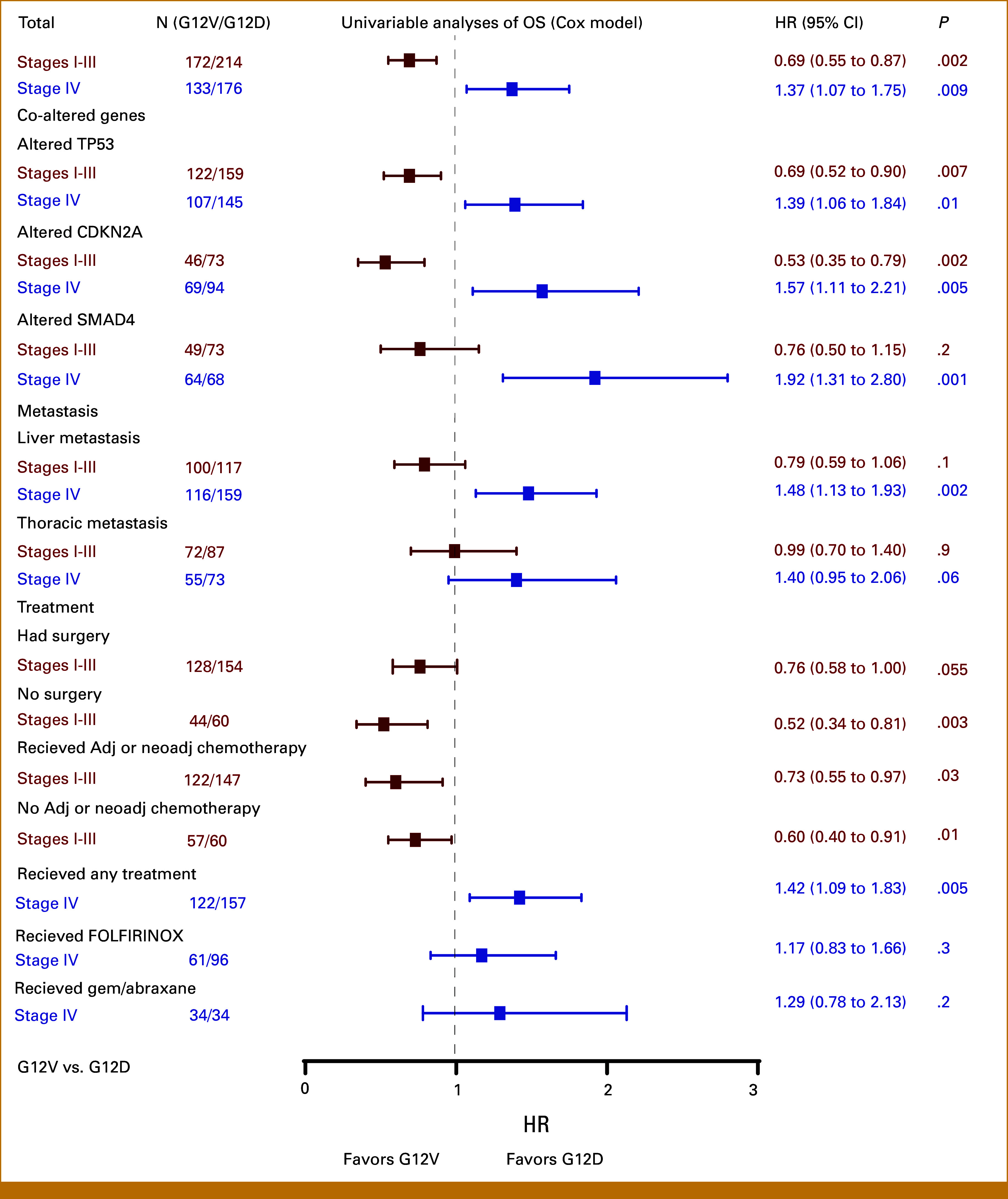
Forest plot of univariable Cox proportional hazards models of OS within subgroups. Univariable analyses of OS in *KRAS* G12V versus G12D status within different stages, alterations in *TP53*, *CDKN2A*, and *SMAD4*, liver and thoracic metastasis, surgical status, adjuvant or neoadjuvant chemotherapy status in stages I to III, and any first-line treatment, first-line FOLFIRINOX status, and first-line gem/abraxane status in stage IV disease. Survival analyses were done using the Cox proportional hazards model, and *P* values were calculated by the Wald test. Adj, adjuvant; gem/abraxane, gemicitabibe/abraxane; HR, hazard ratio; neoadj, neoadjuvant; OS, overall survival.

The G12D and G12V mutations are the most frequent *KRAS* mutations observed in PDAC, occurring in over 70% of cases. Our survival analyses revealed a paradoxical impact of G12V compared with G12D mutation in patients diagnosed with localized versus metastatic disease. We aimed to determine whether this observation was independently associated with *KRAS* codon status or influenced by other factors, such as genomic coalterations in *TP53*, *CDKN2A*, and *SMAD4* genes, patterns of distant metastasis, or treatment regimens. To test the independent associations between G12V and G12D status and survival outcomes, we applied univariable and multivariable Cox regression models (Fig 3 and Data Supplement, Fig S4).

In univariable analysis of patients diagnosed with stage I to III disease, the G12V mutation was associated with longer OS compared with G12D (HR, 0.69 [95% CI, 0.55 to 0.87]; *P* = .002). This association was conserved in the *TP53* (HR, 0.69 [95% CI, 0.52 to 0.90]; *P* = .007) and the *CDKN2A* altered subgroups (HR, 0.53 [95% CI, 0.35 to 0.79]; *P* = .002), as well as across different treatment regimens. In patients with *SMAD4* coalterations, those who eventually developed liver and thoracic metastasis, and in patients who underwent surgery, the G12V mutation was again associated with improved OS, but these differences did not reach statistical significance (all *P* > .05).

Conversely, in univariable analysis of patients diagnosed with metastatic disease, G12V mutation was associated with worse survival compared with the G12D mutation (HR, 1.37 [95% CI, 1.07 to 1.75]; *P* = .009). This trend remained consistent in the presence of alterations in *TP53* (HR, 1.39 [95% CI, 1.06 to 1.84]; *P* = .01), *CDKN2A* (HR, 1.57 [95% CI, 1.11 to 2.21]; *P* = .005), and *SMAD4* (HR, 1.92 [95% CI, 1.31 to 2.80]; *P* = .001), as well as in patients diagnosed with liver metastases (HR, 1.48 [95% CI, 1.13 to 1.93]; *P* = .002) and patients who received any first-line systemic therapy (HR, 1.42 [95% CI, 1.09 to 1.83]; *P* = .005). No statistically significant difference in OS between G12D and G12V mutations were detected within stage IV groups that received specific systemic regimens, possibly because of the limited size of each of these cohorts.

These findings were further confirmed with multivariable analysis using Cox proportional hazards models adjusted for age at diagnosis, sex, race, and treatment center (Data Supplement, Fig S4). The treatment profiles of G12D or G12V mutations are detailed in the Data Supplement (Tables S7-S9).

### Paradoxical Impact of *KRAS* G12V Mutation on Survival Is Associated With Genomic Alterations in Nondriver Genes

**FIG 4.**
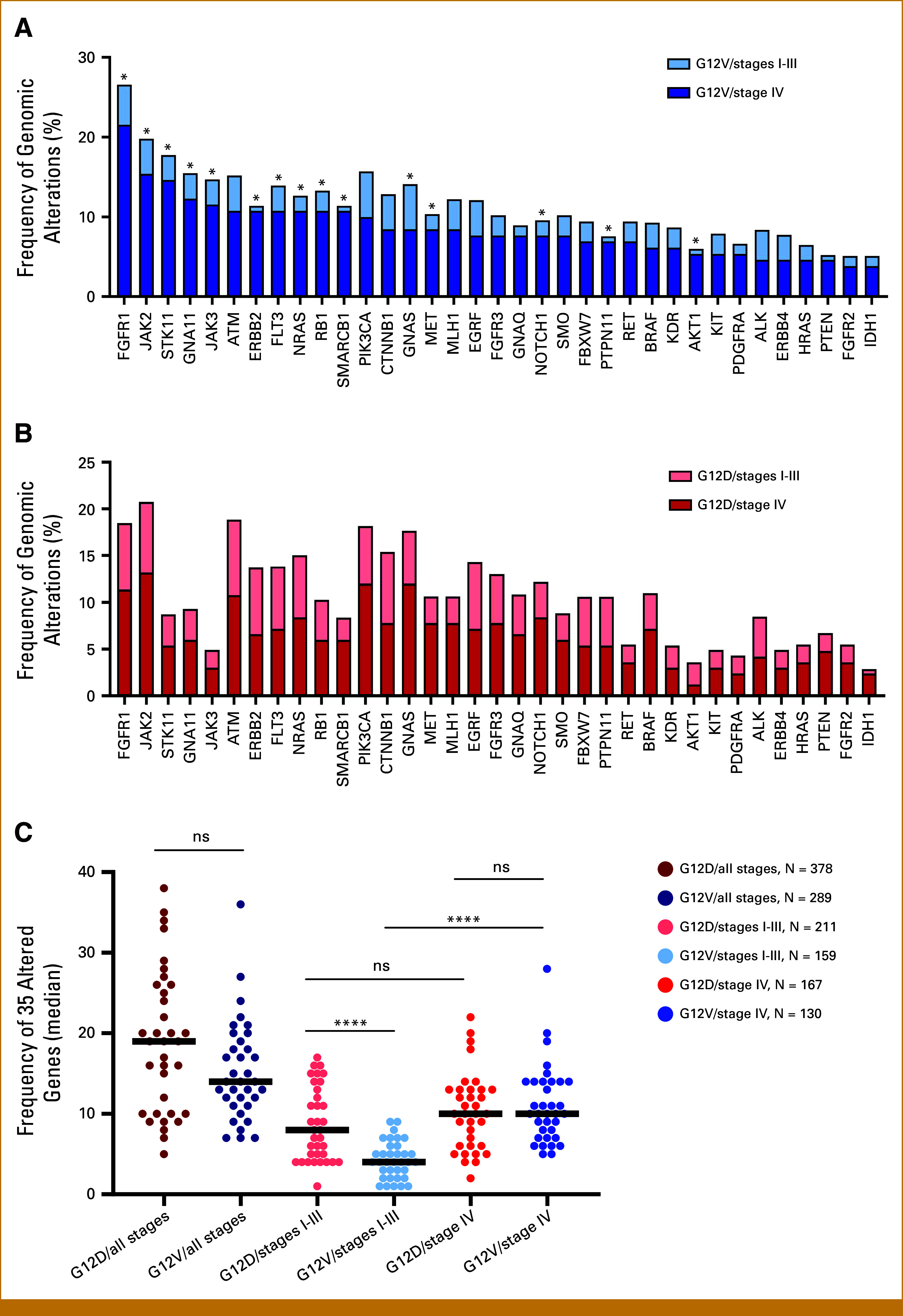
Frequency of genomic alterations in nondriver genes in *KRAS* G12D- and G12V-mutant patients. Stacked bar charts displaying the frequency of genomic alterations including CNVs or mutations of the 35 genes in GENIE cohort (excluding most commonly altered ones including *TP53*, *CDKN2A*, and *SMAD4*) for patients with (A) *KRAS* G12V mutation in stages I to III and IV and (B) *KRAS* G12D mutation in stages I to III and IV. (C) Frequency of overall genomic alterations between *KRAS* G12V and G12D across stages. Each dot corresponds to the frequency of each gene in selected group and horizontal lines regarded as medians. The Wilcoxon rank-sum test was used to compare between groups. FDR correction was applied for multiple comparisons, with a significance threshold of 0.05. *P < .05; ****P < .0001. CNV, copy number variation; FDR, false discovery rate; ns, nonsignificant.

Considering the paradoxical impact of G12V mutation status on PDAC prognosis based on stage at diagnosis, we investigated whether the frequency of CNVs or mutations (referred here as genomic alterations) in the 35 nondriver genes evaluated across all gene panels correlated with different clinical outcomes. Interestingly, the frequency of genomic alterations in 15 of the 35 genes was significantly lower in G12V patients diagnosed with stage I to III disease compared with patients diagnosed with stage IV disease (Fig 4A). However, no significant differences in genomic alterations of nondriver genes were observed between patients with G12D mutation in the stage I to III subgroup versus those in the stage IV subgroup (Fig 4B).

Next, we analyzed the overall genomic alterations across these 35 nondriver genes in the G12D and G12V subgroups by calculating the overall genomic alterations frequencies per group (Fig 4C). Independent of stage, there were no statistically significant differences in the genomic alteration frequency of nondriver genes between patients whose tumors harbored G12D or G12V mutations. We asked whether the frequency of alterations in nondriver genes was different when stratified by stage at diagnosis. Intriguingly, patients with G12V mutations diagnosed with localized disease had the lowest overall genomic alterations, whereas those with G12V diagnosed at advanced-stage had the highest number of genomic alterations (*P* < .001). By contrast, patients with G12D mutation diagnosed with stages I to III versus IV disease did not demonstrate significant differences in nondriver genomic alterations (*P* = .1). Thus, these results suggest that the accumulation of genomic alterations in patients with G12V mutations diagnosed at a later disease stage may explain the paradoxical impact of G12V mutation across localized and advanced stages and might have contributed to the poor prognosis of G12V patients diagnosed with stage IV disease.

### Other Genomic Predictor of Survival

To further elucidate genomic predictors of outcomes in PDAC, we performed univariable and multivariable analyses to assess associations between genomic factors and OS based on stage at diagnosis (Data Supplement, Tables S10 and S11). In patients diagnosed at stage I to III, univariable Cox regression demonstrated significant associations between OS and *KRAS* G12D, G12V, *TP53*, and *CDKN2A* alterations. However, no significant associations were found for *KRAS* G12R, Q61, other *KRAS* mutations, or *SMAD4* alterations. In multivariable Cox analysis, *KRAS* G12D, G12V, and *CDKN2A* alterations remained significant for OS (Data Supplement, Table S10). In patients diagnosed at stage IV, univariable analysis found *KRAS* G12V, *TP53*, and *CDKN2A* alterations were significant predictors of OS. In multivariable analysis, all three genomic alterations remained significantly associated with worse survival (Data Supplement, Table S11). These findings suggest that in patient diagnosed with localized PDAC, *KRAS* G12D and altered *CDKN2A* may serve as predictive biomarkers for reduced OS, while G12V may indicate a more favorable prognosis. By contrast, in patients diagnosed with metastatic disease, *KRAS* G12V and alterations in *TP53* and *CDKN2A* appear to confer the worst survival outcomes.

## DISCUSSION

We used data from AACR's Project GENIE to analyze 1,032 patients with PDAC and specifically test how codon-specific *KRAS* mutations are associated with co-occurring genomic alterations and PDAC outcomes across disease stages. We found that the *KRAS* G12V mutation is associated with significantly longer survival in patients diagnosed with localized PDAC, yet is associated with shorter survival in patients with metastatic disease at the time of diagnosis. Multivariable analyses confirm the paradoxical impact of the G12V mutation across different disease stages, regardless of metastatic patterns, use of different treatment modalities, or alterations in the common tumor suppressors *TP53*, *CDKN2A*, and *SMAD4*. Detailed analyses of additional genomic alterations indicate that changes in frequencies of nondriver genes may explain the paradoxical impact of the G12V mutation on patient outcomes in localized and advanced PDAC. Taken together, our findings highlight the importance of considering codon-specific *KRAS* mutations as key factors in PDAC prognosis based on stage at diagnosis.

To date, studies evaluating the impact of codon-specific *KRAS* mutations in PDAC have produced conflicting results, in part due to the fact that these studies often evaluated patient populations that different based on stage at diagnosis or lacked important clinical annotations.^6,7,14-16,19,24,25^ More recently, studies have emerged that provide detailed depictions of more uniform patient populations. Consistent with our findings, several studies evaluating patients with localized disease have now reported that G12D mutation is associated with significantly worse outcomes.^6,14,15^ Most recently, McIntyre et al compared OS in 397 patients with PDAC diagnosed at stage I to III and found that those diagnosed at stage I with G12D mutation had significantly worse survival compared with patients with G12R and G12V mutations, consistent with the favorable prognosis of G12V in localized PDAC, but not G12R.^16^ This discrepancy for G12R mutation may be due to the limited patient numbers in their cohort and their focus on stage I patients, which had a higher frequency of *KRAS* G12R mutation.

In patients diagnosed with advanced metastatic disease, we found that G12V was associated with worse OS. In alignment with our study, Cheng et al and Norton et al reported that the *KRAS* G12V mutation was associated with poor prognosis in advanced pancreatic cancer.^26,27^ By contrast, Pan et al highlighted the adverse impact of the Q61 mutations on survival in patients with stage IV PDAC,^19^ while another cohort linked G12D to poorer OS in stage IV disease.^7^ Additionally, several studies reported longer OS in patients with metastatic PDAC with the G12R mutation,^27,28^ which does not align with our findings. These discrepancies may reflect the fact that these patient cohorts were sourced from a single institute, as well as limited number of patients in the cohorts.

The G12V mutation has shown variable prognostic significance depending on disease stage in *KRAS*-driven malignancies. In early-stage lung adenocarcinoma, G12V has been associated with better outcomes,^29^ consistent with our finding in localized PDAC. In advanced lung cancer, this mutation has been linked to worse prognosis compared with other *KRAS*-mutant subtypes.^30-32^ Similarly, the G12V has been associated with poor outcomes in metastatic colorectal cancer.^11,33^ These findings in advanced colorectal and lung cancers, along with our data, suggest that G12V may confer unfavorable outcomes in metastatic *KRAS*-driven cancers.

Each *KRAS* mutation has distinct biochemical features that affect guanosine triphosphate hydrolysis, nucleotide exchange, and effector interactions, resulting in varied neoplastic phenotypes.^34-39^ Grimont et al recently reported that G12D more strongly promotes acinar-to-ductal metaplasia and PDAC progression than G12V or G12R. Although transcriptomic profiles were broadly similar, G12D triggered more sustained and pronounced changes over time, aligning with its more aggressive behavior in localized PDAC.^40^

The rapid development and adoption of targeted *KRAS* inhibition through various approaches, including G12C inhibitors,^41,42^ G12D inhibitor,^43^ pan-*KRAS* inhibitor,^44^ pan-RAS inhibitors,^45,46^ and *KRAS* degraders^47^ will almost certainly alter how PDAC is treated across stages. Individual *KRAS* mutations exhibit distinct prognostic implications across different cancer types and stages, which can confound outcome interpretation in early-phase clinical trials. If these differences are not accounted for, they may obscure the true therapeutic effects being evaluated. Therefore, stratifying patients or adjusting analyses based on specific *KRAS* codon variants is critical to accurately assess treatment efficacy.

Notably, the frequency of genomic alterations reported in this study for *KRAS*, *TP53*, *CDKN2A*, and *SMAD4* genes is consistent with other large data sets.^14,19,28,48^ Alterations in each of these tumor suppressor genes were individually associated with worse survival outcomes.^15,16,19,27,49,50^ Although we did not find any association between the codon-specific *KRAS* mutations and alterations in *TP53*, *CDKN2A*, and *SMAD4* as the main driver genes in PDAC, we interestingly uncovered an association between the prognostic impact of *KRAS* G12V in localized and advanced disease stages and differential genomic alterations, primarily driven by CNVs or mutations in 35 nondriver genes in PDAC. However, future studies are required to find any association between codon-specific *KRAS* mutations and whole genomic alterations in PDAC.

Our study's strengths include a large patient cohort from multiple institutions and a comprehensive analysis of diverse clinical variables across all disease stages. However, despite the large sample size, limitations remain. This is a retrospective study and detailed imaging and surgical data, and dynamic variables such as smoking status were not captured. Additionally, the heterogeneity of treatment data restricts us to assess the impact of codon-specific *KRAS* mutations on different chemotherapy, radiation, and investigational treatment responses. Despite these limitations, this study represents the largest effort to evaluate codon-specific *KRAS* mutations across stages of PDAC, leveraging robust clinical annotations to help adjust for confoundings.

## PREPRINT VERSION

Preprint available on https://www.medrxiv.org/content/10.1101/2025.02.03.25321601v1.
